# Self-reported depression and social support are associated with egocentric network characteristics of HIV-infected women of color

**DOI:** 10.1186/s12905-020-00937-3

**Published:** 2020-04-23

**Authors:** Lynne C. Messer, E. Byrd Quinlivan, Adaora Adimora, Katya Roytburd

**Affiliations:** 1 OHSU-PSU School of Public Health, 506 SW Mill St, Portland, OR 97201 USA; 2 Institute for Global Health and Infectious Diseases, University of North Carolina at Chapel Hill, Chapel Hill, NC USA; 3 Center for AIDS Research, University of North Carolina at Chapel Hill, Chapel Hill, NC USA

**Keywords:** HIV, Women, Depression, Social support, Social network

## Abstract

**Background:**

We explore the social network characteristics associated with depressive symptoms and social support among HIV-infected women of color (WOC).

**Methods:**

Network data were collected from 87 HIV-infected WOC at an academic Infectious Disease clinic in the United States (US) south. With validated instruments, interviewers also asked about depressive symptoms, social support, and treatment-specific social support. Linear regression models resulted in beta coefficients and 95% confidence intervals for the relationships among network characteristics, depression, and support provision.

**Results:**

Financial support provision was associated with lower reported depressive symptoms while emotional support provision was associated with increased reported social support. Talking less than daily to the first person named in her network, the primary alter, was associated with a nearly 3-point decrease in reported social support for respondents. Having people in their social network who knew their HIV status was also important.

**Conclusions:**

We found that both functional and structural social network characteristics contributed to perceptions of support by HIV-infected WOC.

## Background

In 2014, women represented 19% of all HIV diagnoses in the United States (US). African American (AA) women comprise only 14% of women in the US, but represent 62% of women newly diagnosed with HIV [1]. HIV-infected women belonging to racial and ethnic minorities (e.g., women of color (WOC)) have lower rates of antiretroroviral therapy (ART) use [2], and adherence to medical appointments [3], and more frequent discontinuations of ART than majority women [4]. Yet ART adherence and continuous HIV care are essential to improve health, increase longevity and reduce HIV transmission [5].

Depression is an important barrier to successful HIV treatment and ART adherence among HIV-infected women of color [6]. African-American women who are infected with HIV and who experience depressive symptoms have low and/or inconsistent use of ART (ART) [7, 8]. In patients with both HIV and substance abuse, mood disorders, including depression, were associated with poor ART adherence unless higher levels of self-efficacy were reported [9].

Successful HIV treatment requires engagement in care, which is facilitated by the presence of general social support from formal and informal networks. In the Antiretroviral Treatment Access Study (ARTAS), even after adjusting for the case management intervention, participants were more likely to remain in care 12 months after enrollment if a member of their social network had tried to help them get HIV treatment [10]. Injection drug users in Baltimore (of whom 33% were women) were more likely to see the same medical provider and receive some medical care in the prior 6 months if their social networks included other women, provided emotional or financial support, or physical assistance [11]. Social network influences are critical.

Social networks can be characterized by their function and structure, where function refers to the type of interaction provided by the network, and structure refers to size, density and overlap [12, 13]. Informed by the Berkman conceptual model [14], whose macro-level social cultural conditions structure social network features, which provide opportunities for micro-level psychological features to impact health through behavioral, psychological and physiologic pathways, we propose that support networks contribute to care engagement by constructing expectations, norms, and pressure to participate in HIV care. Research among HIV-infected men suggests satisfaction with one’s social network and engagement with the AIDS community is associated with more health-supportive coping strategies [15]. Other work indicates HIV-infected people who report receiving emotional and informational support from their network have greater psychological well-being [16]. Numerous studies have shown that social support from formal and informal social networks are important for engaging HIV care [10, 17]. Interestingly, Knowlton et al. [11] found each additional source of support within the network increased the odds of utilizing outpatient services by 24%; the authors suggest that social norms, expectations, and social control contributed to the association between social networks and medical care utilization.

When individuals disclose their HIV status to members of their network, it changes the nature of support that network members can provide. For instance, network members can provide transportation to clinic visits or assistance in picking up HIV medications after HIV disclosure has occurred and Valente et al. [18] found HIV-infected persons were more willing to invite network members to participate in an HIV-vaccine preparedness cohort if the index person had disclosed his/her HIV status to network members. Recent work found HIV-infected women who report feeling loved were less likely to report disclosure stigma [19] while another found early disclosure (within days, instead of a few months after) was associated with lower depression scores among HIV-infected women of color [20]. Among AA and Latino HIV-infected men who have sex with men (MSM) who disclosed their HIV status, the number of persons in their social network was associated with retention in care [21]; Wohl et al’s [21] work suggested that status disclosure was more predictive of care retention than the amount of social support an individual reported receiving. Specifically, among study participants reporting an absence of social support, the use of ART was minimally reduced when study participants had disclosed their HIV status but was significantly reduced if participants had not disclosed their HIV status [22]. For some HIV-infected persons, formal sources of support (HIV care providers and community organizations), rather than the informal network of friends and family, are used to support engagement in HIV care [23].

**Fig. 1:**
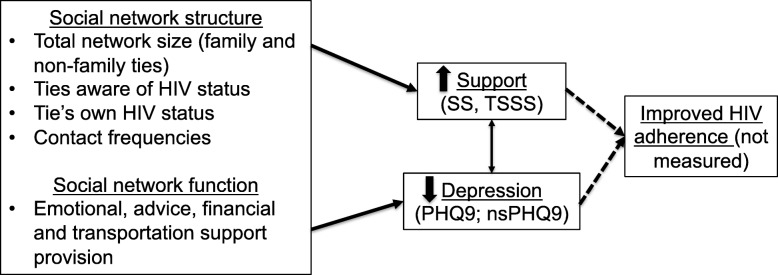
Conceptual model for the relationship between social network attributes, social support, and depression among HIV-infected women of color

Despite the high burden of HIV among WOC, and the seemingly important role played by support networks among HIV-infected individuals, little research has considered the social networks of HIV-infected WOC. In light of the need to further understand the role depression, social support and social networks play as hindering or supporting care engagement among HIV-infected WOC, and in alignment with our conceptual model (Fig. 1) we sought to: 1) Describe the egocentric social network characteristics of a sample of HIV-infected WOC and 2) Evaluate how social network characteristics were associated with depression and social support among these women.

## Methods

### Research setting

The research was conducted in an academic infectious diseases clinic in North Carolina (NC) as part of a broader project called Guide to Healing, which has been described elsewhere [24–27]. The clinic provided HIV care to 1700 HIV infected people in 2010, of whom 58% were African-American (AA), 10% were Hispanic or otherwise not white, which is consistent with NC state epidemiology. Almost all (82%) of the 31% female patients belonged to racial/ethnic minorities, including 69% AA. The clinic’s catchment area includes both rural and urban NC counties.

### Data collection

Serial cross-sectional data were collected as part of an interrupted time series evaluation design of the Guide to Healing project. WOC were eligible to participate in data collection if they met the following criteria: 1) were HIV-infected; 2) had an appointment that day at the clinic; 3) were age 18 or older; and 4) English-speaking. All women of color who showed up for their medical appointment on an interview day were approached to participate in an interview. Social network data were collected as part of the third tier of data collection (2012), the response rate was 58% and participants in the social network analysis represent approximately 25% of the total HIV-infected women of color in the clinic. Validated instruments were used in interviews asking women about depression, trauma, social support, treatment-specific social support and social networks. The survey was read to participants by trained interviewers who entered their responses into netbook computers. Participants received a $25.00 gift card for their time. The University of North Carolina’s Institutional Review Board approved the research protocols.

### Social network variable construction

We used a modified version of the General Social Survey [28] to elicit personal networks. The approach and name generation question was identical to published work, but the characteristics that were solicited for each named network member were modified to answer the research questions outlined in for this analysis. Interviewers asked respondents (the ego) to name the people with whom they have discussed important matters (called alters). Follow-up questions were then asked about the alters, including their relationship with the ego (the choice options included spouse, parent, other family, friend, professional advisor, etc.), frequency of talking with respondent (almost every day to less than once per month), and types of support provided to the respondent (advice, emotional, financial, transportation, other). We also asked if the named alters knew that the respondent had HIV and if s/he (the alter) was also known to the ego to be HIV-infected. Last we asked respondents to indicate if they thought that all, most, only a few, or none of the people they listed in their name generator response knew each other. Counts of named persons by relationship type, number of ties (relationship between ego and alter), type of support provided, frequency of contact with 1st and 2nd-named alters, and awareness of / shared HIV status were generated and used for analyses.

### Outcome variable construction

#### Depression

The PHQ 9 is a validated and reliable 9-item survey designed to screen for potential DSM diagnosable depression [29]. Respondents were asked questions regarding the symptoms experienced over the last 2 weeks and responded on a scale of 0 ( “Not at All”) to 3 (“Nearly every day”). The PHQ 9 was scored by summing the item values (range = 0–27). The physical manifestations of depression, as identified in the PHQ 9 scale, mimic both the symptoms of HIV and the side-effects of HIV medications. Consequently, a psychiatric symptoms subscale (PHQ 4) was also constructed in the identical manner by excluding the questions that asked about physical symptoms of depression. The questions that asked about apathy, depressed mood, guilt, and suicidal ideation were retained and those that asked about insomnia, fatigue, psychomotor changes, appetite changes and concentration difficulties were removed for the psychiatric symptoms subscale PHQ 4.

#### Social support

Perceived emotional and practical social support available from friends and others was captured as a 7-item subset of the Social Support and Activities Scale [30, 31]. Response options ranged from ‘1’ (“Definitely Not”) to 4 (“Definitely Yes”) with a potential range of scores from 7 to 28. The availability of support, care and guidance for HIV care was assessed using the Treatment-specific Social Support Scale (TSSS). TSSS Scale is a modified 12-item subset of the Social Provisions Scale [32]. Responses ranged from 1 (“strongly disagree”) to 4 (“strongly agree”) and were summed to create potential scores from 12 to 48. Each of these social environmental scales were continuous outcomes in our model.

### Covariates

Participants reported their age, employment status, insurance status, years of education and marital status. Age was coded as a continuous variable and employment was coded as a dichotomous variable (full or part-time employment or unemployed). To include an additional indicator of personal resources, insurance status was dichotomized to private insurance and not private insurance while education was coded as a categorical variable (less than 12 years, 12 years, or more than 12 years of education). Marital status was coded as a dichotomized variable (married, unmarried). While marital status is an important descriptive variable for these HIV-infected WOC, it was not included as a covariate in the adjusted models since it is on the causal pathway from social network to depression and social support.

### Data analysis

Means and standard deviations of the continuous variables, counts and percentages of the categorical variables were created. Scale properties were examined, including internal consistency (Cronbach’s alphas) scores, which ranged from 0.90 (treatment-specific social support) to 0.80 (psychiatric symptoms subscale (PHQ 4)). Scale correlations were also explored.

Linear regression was used to assess the relationship between each dependent variable (depression, psychiatric symptoms subscale, social support, and treatment-specific social support) and the various network attributes (count of family ties; count ties providing advice, emotional support, financial support, transportation, respectively; count of network ties who knows HIV status and count of ties who are HIV-infected; total network size; and contact frequency with main and secondary contacts. Models, which had been assessed for linearity assumptions, resulted in beta coefficients and 95% confidence intervals. Adjusted models included continuous age, dichotomous insurance status, and categorical years of education.

## Results

Approximately 90% of the women who participated in the social network data collection were black non-Hispanic, 7% were Native American and the remaining 6% self-identified as Hispanic or other (in non-mutually-exclusive categories, data not shown). Their mean age was 46 years old and approximately 34% had more than 12 years of education (Table 1). Almost all were uninsured or on public insurance, about 30% were employed and most were not married.

**Table 1:** Socio-demographic characteristics and psychosocial status of HIV-infected women of color included in social network analysis (*n* = 87)

Characteristic	Value
Age (mean, standard deviation)	45.5 (10.5)
Age (categorized)	N (%)
< 30 years	6 (6.9)
30–39 years	18 (20.7)
40–49 years	32 (36.8)
50+ years	31 (35.6)
Education years (missing = 1)	
< 12 years	28 (32.6%)
= 12 years	29 (33.7%)
> 12 years	29 (33.7%)
Insurance status (missing = 1)	
Public or uninsured	79 (91.9%)
Private	7 (8.1%)
Employment status (missing = 1)	
Unemployed	60 (69.8%)
Full or part time	26 (30.2%)
Married	
Not married	63 (72.4%)
Married	24 (27.6%)

*Missingness due to one woman not completing the sociodemographic questions on the surey

Women reported relatively high amounts of social support (mean = 24) and treatment-specific social support (mean = 38) (Table 2). The average depression score [7] was in the “mild” depression range (5 to 9 out of 27) for the PHQ 9 screener and the psychiatric symptoms subscale (PHQ 4) was proportionate to the PHQ 9 [3]. The alpha scores ranged from 0.7 (PHQ 4) to 0.9 (treatment-specific social support). Alpha scores for the other scales were higher than 0.8.

**Table 2:** Description of psychosocial scales completed by HIV-infected women of color sample included in social network analysis (*n* = 87)

Scales	Scale score mean (sd)	Theoretical range	Observed range	Chronbach’s alpha
Social support	23.7 (4.0)	7, 28	11, 28	0.84
Treatment-specific support	38.3 (7.3)	12, 48	15, 48	0.88
PHQ 9 depression screener	7.1 (5.7)	0, 27	0, 24	0.82
PHQ 4 psychiatric subscale	3.4 (3.1)	0, 15	0, 12	0.71

A substantial range of family (0 to 5) and non-family ties (0 to 11) were identified (Table 3), with about 84% of reported ties being to family. Spouses (27%) followed by parents, children and / or friends (14.8% each) were the first named alters by respondents. Women also reported that more ties provided advice and emotional support (69 and 73%), than financial and transportation support (43 and 43%, respectively). While many women had ties in their network who knew their HIV status (73%), only 6% of the ties named by the women were also HIV-infected. Most women talked with their first and second named alters almost daily.

**Table 3:** Description of egocentric networks and attributes among HIV-infected women of color represented in the social network analysis (*n* = 87**)

Network variable description	Mean (sd)	% of all ties	Observed range
Count of family ties named	1.9 (1.4)	84.4	0, 5
Count of non-family ties named	1.9 (2.2)	44.4	0, 11
Count of advice ties named	2.3 (1.6)	68.5	0, 5
Count of emotional support* ties named	2.4 (1.5)	72.5	0, 5
Count of financial support ties named	1.3 (1.2)	43.2	0, 5
Count of transportation support ties	1.4 (1.3)	42.5	0, 5
Count of ties who know ego’s HIV status	2.4 (1.6)	73.3	0, 5
Count of ties who are also HIV-infected	0.2 (0.5)	6.0	0, 2
Total network size	3.0 (2.1)	100.0	0, 11
**Connectivity**	**Count**	**Percent**	
No friends, or none know each other	10	12.4	N/A
Only a few friends know each other	28	34.6	N/A
Most friends know each other	26	32.1	N/A
All friends know each other	17	21.0	N/A
**First named alter – contact frequency**	**Count**	**Percent**	**N/A**
Monthly or less	7	8.6	N/A
At least once / week	10	12.4	N/A
Almost daily	64	79.0	N/A
**Second alter – contact frequency**	**Count**	**Percent**	**N/A**
Monthly or less	7	10.9	N/A
At least once / week	14	21.9	N/A
Almost daily	43	67.2	N/A

* by “count of emotional...”, “…financial...” and/or “…transportation ties named” we mean ties that were considered to be providing emotional, financial and / or transportation support

** n = 87 except for count connectivity responses (*n* = 81) and second alter contact frequency (*n* = 80)

While all of the egocentric social network attributes reported by these WOC were associated with lower depression, as indicated by the negative PHQ 9 screening values, the beta coefficients associated with most of these relationships were not distinguishable from zero (Table 4 and Supplemental Table 1). Financial support provision was associated with a one-point reduction in reported depressive symptoms in both adjusted and unadjusted models. One structural network characteristic that appeared to be meaningful was the alter’s HIV status; each additional HIV-infected person in a woman’s social network was associated with 2 fewer points on the PHQ 9 screening tool, even after adjustment for covariates. Talking less than daily with the first named alter was also associated with increased depressive symptoms, but this finding was null following adjustment.

**Table 4:** Unadjusted and adjusted beta coefficients (95% confidence intervals (95% CI)) for association between egocentric social network characteristics, all depression and psychiatric symptoms subscale (PHQ 4) (*n* = 87)

**Depression symptoms (Complete PHQ 9)**				
	Unadjusted models		Adjusted models^a^	
Network characteristic	Coefficient	95% CI	Coefficient	95% CI
Functional network characteristics				
Advice tie count	−0.62	−1.40, 0.17	− 0.57	− 1.36, 0.22
Emotional tie count	− 0.65	− 1.46, 0.15	− 0.58	− 1.41, 0.24
Financial tie count	− 1.15	− 2.14, − 0.15	−1.17	−2.15, − 0.18
Transportation tie count	− 0.51	− 1.49, 0.47	−0.59	− 1.57, 0.38
Structural network characteristics				
Family tie count	−0.69	−1.60, 0.23	−0.72	− 1.66, 0.22
Non-family tie count	−0.47	−1.04, 0.09	− 0.53	−1.08, 0.03
Alter knows ego’s HIV status	−0.63	−1.40, 0.15	− 0.59	− 1.37, 0.19
Alter’s positive HIV status	−2.32	−4.57, − 0.07	− 2.25	−4.49, − 0.01
Total network size	− 0.31	− 0.89, 0.27	− 0.30	−0.69, 0.28
< Daily talk - primary	2.18	0.95, 5.30	1.59	−1.48, 4.65
< Daily talk - secondary	−1.62	−4.47, 1.23	−1.08	−3.88, 1.73
**Psychiatric symptoms subscale (Estimated from 4 PHQ 9 questions)**				
	Unadjusted models		Adjusted models	
	Coefficient	95% CI	Coefficient	95% CI
Functional network characteristics				
Advice tie count	−0.39	− 0.81, 0.03	− 0.35	− 0.78, 0.08
Emotional tie count	− 0.41	− 0.84, − 0.03	−0.36	− 0.81, 0.09
Financial tie count	−0.67	−1.20, − 0.13	−0.65	− 1.19, − 0.11
Transportation tie count	−0.42	− 0.95, 0.10	−0.44	− 0.97, 0.09
Structural network characteristics				
Family tie count	−0.53	− 1.01, − 0.04	−0.53	− 1.04, − 0.01
Non-family tie count	0.14	− 0.45, 0.17	−0.14	− 0.45, 0.17
Alter knows ego’s HIV status	−0.40	− 0.81, − 0.02	−0.36	− 0.79, 0.07
Alter’s positive HIV status	−0.64	−1.88, 0.59	− 0.62	− 1.87, 0.64
Total network size	− 0.22	− 0.54, 0.08	− 0.22	−0.54, 0.10
< Daily talk - primary	1.46	−0.21, 3.12	1.19	−0.49, 2.87
< Daily talk - secondary	−0.92	−2.44, 0.60	− 0.72	− 2.27, 0.83

In social network terms, “ego” is the person responding to the survey and alter is the person who has been named or identified by the ego; ^a^ unadjusted models include only the independent and dependent variables; models adjusted for ego’s continuous age, categorical education, dichotomous insurance status

The pattern of association for the depression screener limited to psychiatric symptoms (PHQ 4) was similar to that observed for the complete PHQ 9 (Table 4); most of the network features were associated with lower reported values on the depression screener. After adjustment for covariates, however, each additional relationship (tie) the respondent thought would provide financial support was associated with a 0.7 point decrease in the psychiatric symptoms subscale. Only one structural characteristic, having family members among the named alters, was associated with lower psychiatric depression scores.

Functional network characteristics were generally associated with higher values of reported social support (Table 5 and Supplemental Table 2), and remained distinguishable from zero following adjustment. Ties that provide advice, emotional, financial and transportation support were associated with increased reported social support. Based on the magnitude of the beta coefficients, few structural network characteristics were associated with social support. The most important influence on reported social support was frequency of contact with the first named alter (the person the respondent named first in her name generator); talking less than daily (between once per week and once per month) was associated with a nearly 3-point decrease in reported social support for respondents. Having a family member among the alters was also important and resulted in a 1-point increase in reported social support.

**Table 5:** Unadjusted and adjusted beta coefficients (95% confidence intervals (95% CI)) for association between egocentric social network characteristics and social support and treatment-specific social support (*n* = 87)

**Social support**				
	Unadjusted models		Adjusted models ^a^	
	Coefficient	95% CI	Coefficient	95% CI
**Functional network characteristics**				
Advice tie count	0.61	0.07, 1.15	0.55	0.03, 1.07
Emotional tie count	0.69	0.14, 1.24	0.69	0.15, 1.23
Financial tie count	1.02	0.34, 1.70	0.91	0.26, 1.56
Transportation tie count	0.85	0.18,1.51	0.84	0.22, 1.48
**Structural network characteristics**				
Family tie count	1.07	0.47, 1.68	1.11	0.52, 1.70
Non-family tie count	0.09	−0.31, 0.49	0.15	−0.60, 0.91
Alter knows ego’s HIV status	0.58	0.04, 1.11	0.50	−0.02, 1.03
Alter’s positive HIV status	−0.60	−2.19, 0.99	−0.69	−2.20, 0.83
Total network size	0.15	− 024, 0.54	0.22	−0.17, 0.61
< Daily talk - primary	−3.23	−5.13, −1.32	−2.87	−4.77, −0.96
< Daily talk - secondary	−0.42	−2.31, 1.46	−0.43	−2.34, 1.47
**Treatment-specific social support**				
	Unadjusted models		Adjusted models	
	Coefficient	95% CI	Coefficient	95% CI
**Functional network characteristics**				
Advice tie count	2.03	1.13, 2.94	1.96	1.03, 2.88
Emotional tie count	2.33	1.43, 3.23	2.38	1.46, 3.31
Financial tie count	2.45	1.26, 3.64	2.30	1.09, 3.50
Transportation tie count	1.69	0.50, 2.88	1.72	0.52, 2.92
**Structural network characteristics**				
Family tie count	2.47	1.42, 3.51	2.54	1.46, 3.63
Non-family tie count	0.37	−0.35, 1.09	0.33	−0.40, 1.05
Alter knows ego’s HIV status	2.20	1.33, 3.08	2.21	1.32, 3.10
Alter’s positive HIV status	−0.15	−3.06, 2.77	0.03	−2.91, 2.97
Total network size	1.26	0.57, 1.94	1.37	0.68, 2.05
< Daily talk – primary	−4.12	−7.88, −0.36	−3.63	−7.44, 0.18
< Daily talk - secondary	−1.11	− 4.73, 2.51	−1.37	− 4.96, 2.22

In social network terms, “ego” is the person responding to the survey and alter is the person who has been named or identified by the ego;^a^ unadjusted models include only the independent and dependent variables; models adjusted for ego’s continuous age, categorical education, dichotomous insurance status

Functional network features were uniformly associated with treatment-specific social support (Table 5); almost all network characteristics were associated with a 2 to 3-point increase in treatment-specific social support, even after adjustment for covariates. Structurally, the count of family ties, having alters who know the ego’s HIV status, and having a larger social network – as indicated by network size, was also associated with higher reported treatment-specific social support. Similar to the findings for social support, talking less than daily to the first named alter was associated with about a 4-point decrease in treatment specific social support.

## Discussion

Social networks have long been understood as a potential risk environment for HIV acquisition and transmission. Social networks may also be an important buffer against depression and a source of social support. This cross-sectional study of HIV-infected WOC in an academic HIV clinic demonstrated that network size and functional characteristics were associated with both decreased depression and increased social support, especially treatment-specific social support.

In this sample of HIV-infected WOC, women named, on average, 4 alters in their social network. While little prior work has assessed network characteristics of HIV-infected WOC, this network size appears similar to others published for HIV-infected African American women (3.3), slightly larger than that reported by HIV-infected African American men who have sex with men (MSM) (2.0), HIV-infected Latino MSM (2.8) and is slightly smaller than prior HIV-infected Latina estimates (3.5) [33]. The size of the network was associated with the availability of treatment specific social support.

Both network function and structure were important across outcomes; functional characteristics were estimated by types of support egos reported receiving from alters. Financial support was associated with reduced depressive symptoms on both the complete PHQ 9 and the psychiatric symptom subscale. Emotional support was also associated with increased general social support and treatment-specific social support. A wider range of functional support (financial, transportation and advice support) was associated with psychiatric depressive symptoms and treatment-specific social support, but not with depressive symptoms or general social support. These findings are consistent with our conceptual model [14, 34], which posits that material resources are an important but incompletely understood aspect of social networks.

Structural network characteristics were also associated with depressive symptoms and reported social support. Depression, measured using the full PHQ 9 scale, was associated with only one structural feature: the HIV-infected status of alters/contacts. This finding suggests that reducing perceived isolation, rather than providing support per se, may be important for reducing depressive symptoms among our sample of HIV-infected WOC.

Frequent contact with the first person named in a woman’s network, another structural feature, was important for reported social support [35]. Reich et al. [35] suggests that effects of social networks on health is dependent upon whether there is an ‘important person’ in an individual’s social network. The findings from the analyses reported here are consistent with others who suggest the important role of having an important person or confidant in supporting mental health and reducing stigma [36]. Prior work has also supported our finding that frequency and quality of interaction with one’s social network may have psychological benefits for HIV-infected women [37]. Interestingly, while most ties are family ties (56%), the count of family ties was only statistically significantly associated with treatment-specific social support. And among the family ties, it appears to be having a spouse that is driving this increase in treatment-specific social support (beta coefficient = 4.60; 95% CI: 1.17, 8.09).

Most women had ties who knew their HIV status (73%), and this HIV status awareness appeared important for receiving more treatment-specific social support and reporting fewer depressive symptoms. This finding is consistent with prior research that disclosure affects willingness to recruit network members to participate in a vaccine preparedness cohort [18], depression scores [20], ART use and reported social support [22]. The observed relationship between the alter knowing the ego’s HIV status and treatment-specific social support make sense, since support can be more effective if the support provider knows what the treatment is for and what constitutes useful or necessary social support. Additionally, the ego may be more likely to request support from someone who is aware of her HIV status. An additional structural characteristic that we were not able to construct with our data, however, was reciprocity, as it was unclear if the HIV-infected alters were the same alters to whom the ego disclosed.

These findings, while promising, are not without limitations. While the sample of 87 women who participated in this survey is not large, it does represent one-quarter of the clinic’s AA female population; these 87 women may not fully represent the population of HIV-infected WOC, however. Further, since our sample included only WOC of one geographic area, the results may not be generalizable to other populations. Because this preliminary analysis did not seek to engage in hypothesis testing, the study power resulting from the 87 participants is adequate for descriptive analyses. Unfortunately, women who do not participate in HIV care may not be well-represented in these analyses. In addition, this analysis was hampered by a lack of detailed adherence data, which means we were not able to assess how network features are associated with treatment adherence, which is a question of public health significance. Finally, these data are cross-sectional, so the causal or directional nature of the relationships between networks and depressive symptoms and social support cannot be determined.

Strengths of this work include our use of validated and widely-used instruments, which facilitates comparison with other research. In this study we also employed a psychiatric symptoms subscale (PHQ 4) to ensure that depressive symptoms were not conflated with HIV symptomatology or medication side-effects. We also used the count of network ties, which facilitates network variable construction and comparison with other research.

## Conclusion

Many WOC living with HIV and AIDS feel isolated and hopeless [25, 27], but the data presented here suggest that both functional and structural characteristics of social networks can be a source of support that ameliorates depression and helps women engage in care. Therefore, understanding women’s existing social networks and how these networks are related to positive and / or negative health behaviors is an important step toward prolonging and improving this population’s quality of life. One important feature of social networks is their modifiability and network interventions, including the use of therapeutic groups, may help keep HIV WOC engaged in care. Future research should evaluate how network ties, an intrinsic community strength, can be harnessed to improve outcomes among women with HIV.

## Supplementary information

**Additional file 1: Table S1.** Overall model statistics (R-squared and Adjusted R-squared) for models presented in Table 4: Unadjusted and adjusted beta coefficients (95% confidence intervals (95% CI)) for association between egocentric social network characteristics, all depression and psychiatric symptoms subscale (PHQ 4) (*n* = 87). **Table S2.** Overall model statistics (R-squared and Adjusted R-squared) for models presented in Table 5: Unadjusted and adjusted beta coefficients (95% confidence intervals (95% CI)) for association between egocentric social network characteristics and social support and treatment-specific social support (*n* = 87).

## Data Availability

The data that support the findings of this study are available on request from the second author (EBQ). The data are not publically available to due them containing personal information that could compromise participant privacy and consent.

## Abbreviations

HIV: Human immunodeficiency virus
WOC: Women of color
US: United States
AA: African American
ART: Antiretroviral therapy
ARTAS: Antiretroviral treatment and access to services
AIDS: Acquired immune deficiency syndrome
MSM: Men who have sex with men
NC: North Carolina
PHQ: Patient health questionnaire
DSM: Diagnostic and statistical manual of mental disorders
TSSS: Treatment-specific social support
95% CI: 95% confidence interval
sd: standard deviation
NA: Not applicable
