# Supplementary material for: Self-reported depression and social support are associated with egocentric network characteristics of HIV-infected women of color
Source: BMC Womens Health. 2020 Apr 23;20:80. doi: 10.1186/s12905-020-00937-3 (PMC7181511; doi:10.1186/s12905-020-00937-3)
Supplement: Supplementary file 1 — Additional file 1: Table S1. Overall model statistics (R-squared and Adjusted R-squared) for models presented in Table 4: Unadjusted and adjusted beta coefficients (95% confidence intervals (95% CI)) for association between egocentric social network characteristics, all depression and psychiatric symptoms subscale (PHQ 4) (n = 87). Table S2. Overall model statistics (R-squared and Adjusted R-squared) for models presented in Table 5: Unadjusted and adjusted beta coefficients (95% confidence intervals (95% CI)) for association between egocentric social network characteristics and social support and treatment-specific social support (n = 87). [file 12905_2020_937_MOESM1_ESM.docx]

ADDITIONAL FILES

SUPPLEMENTARY TABLE 1. Overall model statistics (R-squared and Adjusted R-squared) for models presented in Table 4: Unadjusted and adjusted beta coefficients (95% confidence intervals (95% CI)) for association between egocentric social network characteristics, all depression and psychiatric symptoms subscale (PHQ 4) (n=87).

| **DEPRESSION SYMPTOMS (Complete PHQ 9)** | | | | |
| --- | --- | --- | --- | --- |
|  | UNADJUSTED MODELS | | ADJUSTED MODELS^a^ | |
| Network characteristic | Coefficient | 95% CI | Coefficient | 95% CI |
| FUNCTIONAL NETWORK CHARACTERISTICS | | | | |
| Advice tie count | -0.62 | -1.40, 0.17 | -0.57 | -1.36, 0.22 |
|  | R-squared = 0.03  Adj R-squared = 0.02 | | R-squared = 0.12  Adj R-squared = 0.06 | |
| Emotional tie count | -0.65 | -1.46, 0.15 | -0.58 | -1.41, 0.24 |
|  | R-squared = 0.03  Adj R-squared = 0.02 | | R-squared = 0.12  Adj R-squared = 0.06 | |
| Financial tie count | -1.15 | -2.14, -0.15 | -1.17 | -2.15, -0.18 |
|  | R-squared = 0.06  Adj R-squared = 0.05 | | R-squared = 0.15  Adj R-squared = 0.10 | |
| Transportation tie count | -0.51 | -1.49, 0.47 | -0.59 | -1.57, 0.38 |
|  | R-squared = 0.01  Adj R-squared = 0.001 | | R-squared = 0.11  Adj R-squared = 0.06 | |
| STRUCTURAL NETWORK CHARACTERISTICS | | | | |
| Family tie count | -0.69 | -1.60, 0.23 | -0.72 | -1.66, 0.22 |
|  | R-squared = 0.03  Adj R-squared = 0.01 | | R-squared = 0.12  Adj R-squared = 0.07 | |
| Non-family tie count | -0.47 | -1.04, 0.09 | -0.53 | -1.08, 0.03 |
|  | R-squared = 0.03  Adj R-squared = 0.02 | | R-squared = 0.13  Adj R-squared = 0.08 | |
| Alter knows ego’s HIV status | -0.63 | -1.40, 0.15 | -0.59 | -1.37, 0.19 |
|  | R-squared = 0.03  Adj R-squared = 0.02 | | R-squared = 0.12  Adj R-squared = 0.06 | |
| Alter’s positive HIV status | -2.32 | -4.57, -0.07 | -2.25 | -4.49, -0.01 |
|  | R-squared = 0.05  Adj R-squared = 0.04 | | R-squared = 0.14  Adj R-squared = 0.08 | |
| Total network size | -0.31 | -0.89, 0.27 | -0.30 | -0.69, 0.28 |
|  | R-squared = 0.03  Adj R-squared = 0.01 | | R-squared = 0.12  Adj R-squared = 0.06 | |
| < Daily talk - primary | 2.18 | 0.95, 5.30 | 1.59 | -1.48, 4.65 |
|  | R-squared = 0.01  Adj R-squared = 0.001 | | R-squared = 0.13  Adj R-squared = 0.07 | |
| < Daily talk - secondary | -1.62 | -4.47, 1.23 | -1.08 | -3.88, 1.73 |
|  | R-squared = 0.004  Adj R-squared = 0.001 | | R-squared = 0.14  Adj R-squared = 0.07 | |
| **PSYCHIATRIC SYMPTOMS SUBSCALE (Estimated from 4 PHQ 9 questions)** | | | | |
|  | UNADJUSTED MODELS | | ADJUSTED MODELS | |
|  | Coefficient | 95% CI | Coefficient | 95% CI |
| FUNCTIONAL NETWORK CHARACTERISTICS | | | | |
| Advice tie count | -0.39 | -0.81, 0.03 | -0.35 | -0.78, 0.08 |
|  | R-squared = 0.04  Adj R-squared = 0.03 | | R-squared = 0.09  Adj R-squared = 0.03 | |
| Emotional tie count | -0.41 | -0.84, -0.03 | -0.36 | -0.81, 0.09 |
|  | R-squared = 0.07  Adj R-squared = 0.04 | | R-squared = 0.09  Adj R-squared = 0.03 | |
| Financial tie count | -0.67 | -1.20, -0.13 | -0.65 | -1.19, -0.11 |
|  | R-squared = 0.07  Adj R-squared = 0.06 | | R-squared = 0.12  Adj R-squared = 0.06 | |
| Transportation tie count | -0.42 | -0.95, 0.10 | -0.44 | -0.97, 0.09 |
|  | R-squared = 0.03  Adj R-squared = 0.02 | | R-squared = 0.09  Adj R-squared = 0.03 | |
| STRUCTURAL NETWORK CHARACTERISTICS | | | | |
| Family tie count | -0.53 | -1.01, -0.04 | -0.53 | -1.04, -0.01 |
|  | R-squared = 0.05  Adj R-squared = 0.04 | | R-squared = 0.10  Adj R-squared = 0.05 | |
| Non-family tie count | 0.14 | -0.45, 0.17 | -0.14 | -0.45, 0.17 |
|  | R-squared = 0.01  Adj R-squared = 0.001 | | R-squared = 0.07  Adj R-squared = 0.01 | |
| Alter knows ego’s HIV status | -0.40 | -0.81, -0.02 | -0.36 | -0.79, 0.07 |
|  | R-squared = 0.04  Adj R-squared = 0.03 | | R-squared = 0.09  Adj R-squared = 0.03 | |
| Alter’s positive HIV status | -0.64 | -1.88, 0.59 | -0.62 | -1.87, 0.64 |
|  | R-squared = 0.01  Adj R-squared = 0.001 | | R-squared = 0.07  Adj R-squared = 0.01 | |
| Total network size | -0.22 | -0.54, 0.08 | -0.22 | -0.54, 0.10 |
|  | R-squared = 0.03  Adj R-squared = 0.01 | | R-squared = 0.08  Adj R-squared = 0.01 | |
| < Daily talk - primary | 1.46 | -0.21, 3.12 | 1.19 | -0.49, 2.87 |
|  | R-squared = 0.02  Adj R-squared = 0.001 | | R-squared = 0.09  Adj R-squared = 0.03 | |
| < Daily talk - secondary | -0.92 | -2.44, 0.60 | -0.72 | -2.27, 0.83 |
|  | R-squared = 0.006  Adj R-squared = 0.001 | | R-squared = 0.08  Adj R-squared = 0.01 | |

Notes: in social network terms, “ego” is the person responding to the survey and alter is the person who has been named or identified by the ego; ^a^ unadjusted models include only the independent and dependent variables; models adjusted for ego’s continuous age, categorical education, dichotomous insurance status

SUPPLEMENTARY TABLE 2. Overall model statistics (R-squared and Adjusted R-squared) for models presented in Table 5: Unadjusted and adjusted beta coefficients (95% confidence intervals (95% CI)) for association between egocentric social network characteristics and social support and treatment-specific social support (n=87).

| **SOCIAL SUPPORT** | | | | |
| --- | --- | --- | --- | --- |
|  | UNADJUSTED MODELS | | ADJUSTED MODELS ^a^ | |
|  | Coefficient | 95% CI | Coefficient | 95% CI |
| FUNCTIONAL NETWORK CHARACTERISTICS | | | | |
| Advice tie count | 0.61 | 0.07, 1.15 | 0.55 | 0.03, 1.07 |
|  | R-squared = 0.06  Adj R-squared = 0.05 | | R-squared = 0.15  Adj R-squared = 0.10 | |
| Emotional tie count | 0.69 | 0.14, 1.24 | 0.69 | 0.15, 1.23 |
|  | R-squared = 0.07  Adj R-squared = 0.06 | | R-squared = 0.17  Adj R-squared = 0.12 | |
| Financial tie count | 1.02 | 0.34, 1.70 | 0.91 | 0.26, 1.56 |
|  | R-squared = 0.10  Adj R-squared = 0.08 | | R-squared = 0.18  Adj R-squared = 0.13 | |
| Transportation tie count | 0.85 | 0.18,1.51 | 0.84 | 0.22, 1.48 |
|  | R-squared = 0.07  Adj R-squared = 0.06 | | R-squared = 0.18  Adj R-squared = 0.13 | |
| STRUCTURAL NETWORK CHARACTERISTICS | | | | |
| Family tie count | 1.07 | 0.47, 1.68 | 1.11 | 0.52, 1.70 |
|  | R-squared = 0.13  Adj R-squared = 0.12 | | R-squared = 0.24  Adj R-squared = 0.19 | |
| Non-family tie count | 0.09 | -0.31, 0.49 | 0.15 | -0.60, 0.91 |
|  | R-squared = 0.002  Adj R-squared = 0.001 | | R-squared = 0.11  Adj R-squared = 0.05 | |
| Alter knows ego’s HIV status | 0.58 | 0.04, 1.11 | 0.50 | -0.02, 1.03 |
|  | R-squared = 0.05  Adj R-squared = 0.04 | | R-squared = 0.14  Adj R-squared = 0.09 | |
| Alter’s positive HIV status | -0.60 | -2.19, 0.99 | -0.69 | -2.20, 0.83 |
|  | R-squared = 0.01  Adj R-squared = 0.001 | | R-squared = 0.11  Adj R-squared = 0.06 | |
| Total network size | 0.15 | -024, 0.54 | 0.22 | -0.17, 0.61 |
|  | R-squared = 0.02  Adj R-squared = 0.001 | | R-squared = 0.12  Adj R-squared = 0.06 | |
| < Daily talk - primary | -3.23 | -5.13, -1.32 | -2.87 | -4.77, -0.96 |
|  | R-squared = 0.11  Adj R-squared = 0.10 | | R-squared = 0.18  Adj R-squared = 0.13 | |
| < Daily talk - secondary | -0.42 | -2.31, 1.46 | -0.43 | -2.34, 1.47 |
|  | R-squared = 0.04  Adj R-squared = 0.03 | | R-squared = 0.12  Adj R-squared = 0.05 | |
| **TREATMENT-SPECIFIC SOCIAL SUPPORT** | | | | |
|  | UNADJUSTED MODELS | | ADJUSTED MODELS | |
|  | Coefficient | 95% CI | Coefficient | 95% CI |
| FUNCTIONAL NETWORK CHARACTERISTICS | | | | |
| Advice tie count | 2.03 | 1.13, 2.94 | 1.96 | 1.03, 2.88 |
|  | R-squared = 0.19  Adj R-squared = 0.18 | | R-squared = 0.23  Adj R-squared = 0.18 | |
| Emotional tie count | 2.33 | 1.43, 3.23 | 2.38 | 1.46, 3.31 |
|  | R-squared = 0.24  Adj R-squared = 0.23 | | R-squared = 0.29  Adj R-squared = 0.24 | |
| Financial tie count | 2.45 | 1.26, 3.64 | 2.30 | 1.09, 3.50 |
|  | R-squared = 0.16  Adj R-squared = 0.15 | | R-squared = 0.20  Adj R-squared = 0.15 | |
| Transportation tie count | 1.69 | 0.50, 2.88 | 1.72 | 0.52, 2.92 |
|  | R-squared = 0.09  Adj R-squared = 0.07 | | R-squared = 0.14  Adj R-squared = 0.09 | |
| STRUCTURAL NETWORK CHARACTERISTICS | | | | |
| Family tie count | 2.47 | 1.42, 3.51 | 2.54 | 1.46, 3.63 |
|  | R-squared = 0.21  Adj R-squared = 0.20 | | R-squared = 0.26  Adj R-squared = 0.21 | |
| Non-family tie count | 0.37 | -0.35, 1.09 | 0.33 | -0.40, 1.05 |
|  | R-squared = 0.01  Adj R-squared = 0.003 | | R-squared = 0.07  Adj R-squared = 0.01 | |
| Alter knows ego’s HIV status | 2.20 | 1.33, 3.08 | 2.21 | 1.32, 3.10 |
|  | R-squared = 0.23  Adj R-squared = 0.22 | | R-squared = 0.28  Adj R-squared = 0.23 | |
| Alter’s positive HIV status | -0.15 | -3.06, 2.77 | 0.03 | -2.91, 2.97 |
|  | R-squared = 0.001  Adj R-squared = 0.000 | | R-squared = 0.06  Adj R-squared = 0.001 | |
| Total network size | 1.26 | 0.57, 1.94 | 1.37 | 0.68, 2.05 |
|  | R-squared = 0.03  Adj R-squared = 0.01 | | R-squared = 0.05  Adj R-squared = 0.01 | |
| < Daily talk – primary | -4.12 | -7.88, -0.36 | -3.63 | -7.44, 0.18 |
|  | R-squared = 0.05  Adj R-squared = 0.04 | | R-squared = 0.10  Adj R-squared = 0.04 | |
| < Daily talk - secondary | -1.11 | -4.73, 2.51 | -1.37 | -4.96, 2.22 |
|  | R-squared = 0.06  Adj R-squared = 0.04 | | R-squared = 0.17  Adj R-squared = 0.10 | |

Notes: in social network terms, “ego” is the person responding to the survey and alter is the person who has been named or identified by the ego; ^a^ unadjusted models include only the independent and dependent variables; models adjusted for ego’s continuous age, categorical education, dichotomous insurance status
